# Clinical presentation, management, and outcomes of aortic endograft infections: a retrospective descriptive study

**DOI:** 10.1177/20499361261465761

**Published:** 2026-07-16

**Authors:** Haitham Alaithan, Sarwat Khalil, Gustavo Oderich, Larry M. Baddour, Muhammad Rizwan Sohail

**Affiliations:** Section of Infectious Diseases, Department of Medicine, Baylor College of Medicine, 7200 Cambridge St., STE 8B, Houston, TX 77030, USA; Department of Internal Medicine, College of Medicine, Imam Abdulrahman Bin Faisal University, Dammam 34212, Saudi Arabia; Section of Infectious Diseases, Department of Medicine, Baylor College of Medicine, Houston, TX, USA; Division of Vascular Surgery and Endovascular Therapy, Baylor College of Medicine, Houston, TX, USA; Division of Infectious Diseases, Department of Internal Medicine, Mayo Clinic College of Medicine and Science, Rochester, MN, USA; Section of Infectious Diseases, Department of Medicine, Baylor College of Medicine, 7200 Cambridge St., STE 8B, Houston, TX 77030, USA

**Keywords:** aortic endograft infection, diagnosis, graft, management, outcomes, suppression

## Abstract

**Background:**

Aortic endograft infection (AEGI) is a rare complication of abdominal endovascular aortic repair (EVAR) and thoracic endovascular aortic repair (TEVAR). Surgical explantation with reconstruction is recommended, but data on medical management remain limited. We present our institutional experience with AEGI.

**Objective:**

To describe the clinical presentation, microbiology, management strategies, and outcomes of patients with AEGI, with the aim of addressing current gaps in the literature and informing future research to improve patient outcomes.

**Design:**

Retrospective descriptive study.

**Methods:**

Adult patients admitted with AEGI at our institution between January 2017 and August 2024 were identified using ICD-10 codes and confirmed using the Management of Aortic Graft Infection Collaboration (MAGIC) diagnostic criteria. Clinical, microbiological, radiographic, treatment, and outcome data were collected and analyzed.

**Results:**

We screened 517 patients, and 46 met AEGI criteria (EVAR 54%, TEVAR 46%). Most (63%) presented with late-onset infections (⩾3 months). Fever (54%) and sepsis (48%) were common presenting symptoms. Computed tomography (CT) (85%) was the primary imaging modality. Blood cultures were positive in 54% of the cases, with *Staphylococcus aureus* being the most common monomicrobial pathogen (24%), and 24% of cases were polymicrobial. The majority (67%) received combined medical and surgical therapy. Chronic antibiotic suppressive therapy was used in 34 (74%), most often tetracyclines (33%). Overall, treatment success rates were similar between surgical and medical groups (58% vs 60%, *p* = 0.250) with comparable mortality. However, relapses were more frequent in the medical-only group (33% vs 16%, *p* = 0.185).

**Conclusion:**

Most patients presented with late-onset AEGI. Fever was absent in nearly half of cases, and blood cultures were positive in only 54%. Overall, surgical and medical therapy had similar success, though relapses were more frequent in those treated medically alone.

## Plain language summary

Aortic stent graft infections are rare but serious and may occur without fever; surgery and antibiotics have similar success, but relapse is more common with antibiotics alone

Aortic endografts (also called stent grafts) are devices placed inside the aorta to treat aneurysms or other aortic diseases without open surgery. In rare cases, these devices can become infected. Although uncommon, these infections are serious and can lead to severe illness or death if not treated properly. Diagnosing an infection of an aortic stent graft can be difficult. Patients do not always have typical signs of infection, such as fever or bacteria in the blood. Because the symptoms can be subtle, doctors often need a high level of suspicion and must rely on imaging studies, laboratory tests, and clinical judgment to make the diagnosis. Treatment usually involves either surgery to remove the infected graft, along with antibiotic therapy, or antibiotic therapy alone when surgery is not possible or considered too risky. Surgery is a major operation and may not be suitable for all patients, especially those with other serious medical conditions. In this study, we evaluated patients with aortic endograft infections to better understand how they present and how well different treatments work. We found that both surgical treatment combined with antibiotics and antibiotic treatment alone achieved similar overall success in controlling infection. However, patients treated with antibiotics alone were more likely to experience a relapse of the infection. These findings highlight that aortic endograft infections are challenging to diagnose and treat. Clinicians should consider this diagnosis even when typical signs of infection are absent. While surgery remains an important treatment option, antibiotics alone may still control infection in some patients, although the risk of recurrence appears to be higher.

## Introduction

Endovascular aneurysm repair is a minimally invasive procedure that involves the placement of a stent, an endograft, within the aorta, either thoracic endovascular aortic repair (TEVAR) or abdominal endovascular aortic repair (EVAR), to address various aortic pathologies. In the past, EVAR was primarily used for high-risk patients who were not suitable candidates for open surgery. However, it has increasingly replaced open surgical repairs as a result of being a minimally invasive procedure and subsequently reduced risk of post-operative complications such as infections, as well as reduced perioperative 30-day mortality rates (0.5%–2.9% vs 3.0%–5.1%).^1–3^ Aortic endograft infection (AEGI) is a rare complication with reported incidence rate between 0.5% and 5%.^4,5^ However, despite its rarity, it poses significant clinical challenges in management and is associated with high (25%–38%) mortality.^6,7^

AEGI management guidelines recommend complete surgical explantation and in situ reconstruction for patients who are suitable surgical candidates.^8,9^ Even in surgically managed patients, chronic antimicrobial suppressive (CAS) therapy is frequently employed because of placement of a new graft at the site of active infection. Data regarding the medical management of AEGI are limited, particularly in areas such as optimal antimicrobial selection, duration of induction therapy, long-term CAS therapy, and its associated outcomes. In this study, we report our institutional experience with AEGI at Baylor St. Luke’s Medical Center (BSLMC) in Houston, TX (USA), a tertiary care referral center with a large vascular surgery program. Our objective is to fill the current gaps in the literature and drive further research to enhance patient outcomes in AEGI management.

## Materials and methods

### Study design and eligibility criteria

We conducted a retrospective review of all adults (⩾18 years of age) who were admitted from January 1st, 2017 to August 31st, 2024 at BSLMC with suspected AEGI. Potential cases were identified through an electronic medical record (EMR) query for encounters assigned any of the following World Health Organization (WHO) ICD-10 codes T82.7 (infection and inflammatory reaction due to other cardiac and vascular devices, implants or grafts), Z86.79 (personal history of other diseases of the circulatory system), and Z95 (presence of cardiac and vascular implants and grafts). These codes were used as screening identifiers and were not required to occur in combination. All records identified through this search were subsequently reviewed manually.

Only patients meeting the Management of Aortic Graft Infection Collaboration (MAGIC) criteria definition of a diagnosed infection were included, defined as the presence of one major criterion plus at least one additional criterion (major or minor) from a different diagnostic category.^10^ Patients younger than 18 years of age, those admitted outside the study period, and those who did not meet the MAGIC criteria for a diagnosed AEGI, including patients with only suspected infection or insufficient clinical, microbiologic, or imaging evidence to confirm the diagnosis, were excluded.

### AEGI infection definitions

Early AEGI was defined as infection occurring <3 months from endograft implantation, and late AEGI as infection occurring ⩾3 months after implantation. The 3-month threshold was pre-specified.

Additional definitions included the following: sepsis, defined as the presence of suspected infection in addition to at least two systemic inflammatory response (SIRS) criteria; temperature >38°C or <36°C, heart rate >90 beats per minute, respiratory rate >20 breath per minute, or white blood cell count of >12 × 10^9^/L or <4 × 10^9^/L.

Complete resection was defined as full removal of the infected endograft; partial graft resection referred to incomplete removal of graft material, such as partial stent extraction or removal of a prior open surgical graft while retaining endovascular stent components. Curative antimicrobial was defined as antimicrobial treatment with the potential to eradicate the infection, while suppressive antimicrobial treatment was defined as antimicrobial treatment intended to suppress the infection, without the potential to eradicate the infection.

Treatment success was defined as the absence of the following events during the available follow-up period: reoperation at the vascular graft site, infection-related death, persistent infection, or recurrent infection. Relapse was defined as recurrence of bloodstream infection or clinical manifestations attributable to the original AEGI episode. Mortality was defined as all-cause death occurring during the index hospitalization or documented in the EMR during the available follow-up period.

The follow-up period was defined as the time from index admission to death or the end of the study period, whichever occurred first. Given the retrospective design and the fact that BSLMC serves as a tertiary referral center with a substantial proportion of patients transferred from outside institutions and subsequently followed elsewhere, uniform 1-year follow-up was not consistently available. Therefore, outcome definitions were based on events documented during the available follow-up rather than the 1-year consensus definition of cure proposed in a recent Delphi census document.^11^

### Data collection

Clinical data were extracted from electronic medical records and managed using REDCap^®^,^12,13^ hosted by Baylor College of Medicine (BCM). Collected variables included patient-specific data (demographics and comorbidities), infection-specific data (clinical presentation at index admission, diagnostic modalities, and microbiology findings), treatment details, and clinical outcomes.

Plasma microbial cell-free DNA sequencing (Karius^®^, Karius Inc., Redwood City, CA, USA) was performed at the discretion of the treating clinicians. The assay uses next-generation sequencing to detect and quantify microbial cell-free DNA (mcfDNA) in plasma, enabling identification of bacterial, viral, fungal, and parasitic pathogens. Test results were abstracted from the medical record when available and included in the microbiologic data analysis. For patients who were referred from external institutions, medical records were reviewed when available; otherwise, data at the time of presentation to BSLMC were considered the index admission. Patients with multiple admissions for recurrent AEGI were analyzed as a single data entry, with the first qualifying admission during the study period designated as the index admission. Two patients had thoracoabdominal endografts involving both thoracic and abdominal segments; for analytic purposes, these cases were categorized according to the primary site of infection based on radiographic findings. In both cases, imaging demonstrated infection involving the thoracic segment, and they were therefore classified within the TEVAR infection group.

### Statistical analysis

Given the retrospective design of this study and the rarity of aortic endograft infection, no sample size calculation or power analysis was performed before the study. A descriptive analysis was performed to calculate frequencies, tendencies, and variability in the data. Categorical variables were compared using the Chi-square or Fisher’s exact test, as appropriate, while continuous variables were analyzed using the Wilcoxon rank-sum test. A two-sided *p* value of <0.05 was considered statistically significant. Survival analysis was performed with the Kaplan-Meier method. All statistical analyses were performed using Stata version 17 (StataCorp, College Station, TX, USA).

### Reporting guideline:

The reporting of this study conforms to the Strengthening the Reporting of Observational Studies in Epidemiology (STROBE) statement for observational studies. The completed STROBE checklist is provided as a Supplemental File.^14^

## Results

### Patient demographics and comorbidities

A total of 510 patients were screened, of whom 46 patients met the study definition for AEGI. Table 1 displays the demographics and comorbidities of the cohort. Of the 46 patients, 25 (54%) had EVAR infection, and 21 (46%) had TEVAR infection. The median age was 69 years (IQR 62–75) in the EVAR group and 62 years (IQR 58–65) in the TEVAR group (*p* = 0.004). Overall, the majority of patients were white (78%). The most common comorbidity was hypertension, present in 37 patients (80%), followed by obesity in 27 (59%), and diabetes mellitus in 7 (15%). Thirteen patients had prosthetic or foreign material devices at the time of infection, including prosthetic heart valve (*N* = 4), prosthetic joints (*N* = 4), cardiac device (pacemaker or ICD; *N* = 2), spine hardware (*N* = 2), and other vascular graft (*N* = 1). Charlson Comorbidity Index score was similar between both groups, with a median of 4.

**Table 1.** Patients’ demographics and characteristics.

Baseline characteristics	EVAR, *N* = 25	TEVAR, *N* = 21	Total, *N* = 46	*p* Value
Age, years, median (IQR)	69 (62–75)	62 (58–65)	64 (61–74)	0.004
Female sex	6 (24%)	8 (38%)	14 (30%)	0.349
Race				0.411
White	21 (84%)	15 (71%)	36 (78%)	
African American	4 (16%)	5 (24%)	9 (20%)	
Asian	0	1 (5%)	1 (2%)	
Comorbidities				
Hypertension	20 (80%)	17 (81%)	37 (80%)	0.935
Diabetes Mellitus	6 (24%)	1 (5%)	7 (15%)	0.106
Obesity	16 (64%)	11 (52%)	27 (59%)	0.425
Presence of Prosthesis	8 (32%)	5 (24%)	13 (28%)	0.539
Charlson comorbidity index score, SD	4 ± 1	4 ± 2	4 ± 2	0.130

Data presented as number (%), unless otherwise indicated.

a Prosthesis included prosthetic heart valve (*n* = 4), prosthetic joints (*n* = 4), cardiac device (pacemaker or ICD; *n* = 2), spine hardware (*n* = 2), and other vascular graft (*n* = 1).

EVAR, endovascular aortic repair; IQR, interquartile range; SD, standard deviation; TEVAR, thoracic endovascular aortic repair.

### Clinical presentation

**Figure 1.**
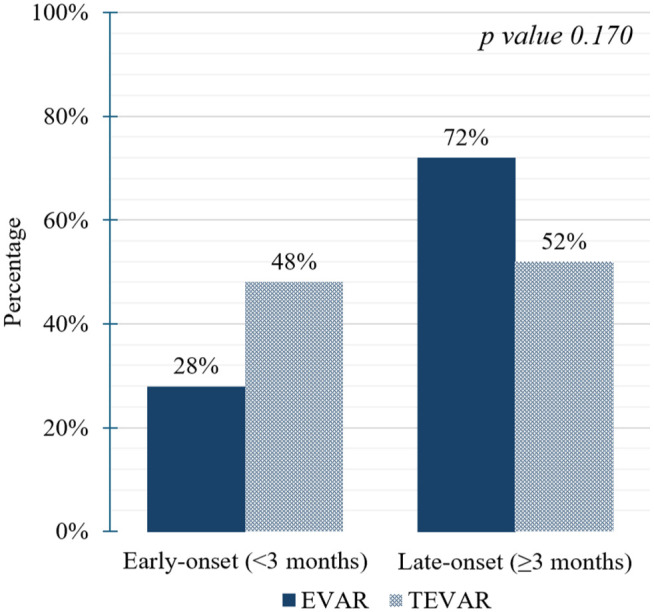
Onset of infection based on the location of the endograft.

Among the 46 patients, 29 (63%) patients presented with a late-onset infection (⩾3 months after endograft placement), while only 17 (37%) had early-onset infection (<3 months). Early-onset infection was observed in 7 (28%) patients in the EVAR group, and 10 (48%) in the TEVAR group. Late-onset infection was observed in 18 (72%) patients in the EVAR group, and 11 (52%) in the TEVAR group (Figure 1). There was no statistically significant difference in the infection onset between groups (*p* = 0.170). The median duration from onset of symptoms to diagnosis was 14 days (IQR 5–39).

Overall, fever was documented in 25 (54%) patients. Sepsis was more frequently observed in the TEVAR group (*N* = 13, 62%) compared to the EVAR group (*N* = 9, 36%), with no statistically significant difference (*p* = 0.080). Abdominal pain was significantly more common in the EVAR group (56% vs 14%, *p* = 0.004), while chest pain was only observed in the TEVAR group (7%). Local findings including surgical site erythema, tenderness, open wound, drainage, or sinus tract were more often observed in the TEVAR group compared to EVAR (26% vs 4%, *p* = 0.021). Bleeding, including hematochezia or melena, was seen in 5 (20%) patients in the EVAR group, and hemoptysis in 1 (5%) patient in the TEVAR group. Nonspecific symptoms included nausea and vomiting (20%), back pain (17%), and malaise (15%), with no significant differences between groups. One patient (4%) in the EVAR group presented with acute limb ischemia.

At the time of diagnosis, 4 (9%) patients had evidence of distant infection, including discitis/osteomyelitis of the spine (*N* = 2), splenic infarction (*N* = 1), and a patient with both septic arthritis (*N* = 1) and a kidney infarction (*N* = 1). The mean white blood cells (WBC) count was 12.7 × 10^9^ cells per liter (cells/L) with standard deviation (SD ± 5.0). Acute kidney injury was documented in 10 patients (22%). A summary of the clinical presentation is provided in Table 2.

**Table 2.** Clinical presentation at index admission.

Clinical characteristics	EVAR, *N* = 25	TEVAR, *N* = 21	Total, *N* = 46	*p* Value
Onset of infection				0.170
Early-onset (<3 months)	7 (28%)	10 (48%)	17 (37%)	
Late-onset (⩾3 months)	18 (72%)	11 (52%)	29 (63%)	
Time from symptoms to diagnosis, days, median (IQR)	11 (5–26)	18 (4–39)	14 (5–39)	0.683
Clinical signs and symptoms				
Fever	12 (48%)	13 (61%)	25 (54%)	0.346
Sepsis^a^	9 (36%)	13 (62%)	22 (48%)	0.080
Abdominal pain	14 (56%)	3 (14%)	17 (37%)	0.004
Nausea or vomiting	5 (20%)	4 (19%)	9 (20%)	0.935
Back pain	6 (24%)	2 (10%)	9 (17%)	0.197
Malaise	4 (16%)	3 (14%)	7 (15%)	0.872
Presence of local findings^b^	1 (4%)	6 (26%)	7 (15%)	0.021
Bleeding^c^	5 (20%)	1 (5%)	6 (13%)	0.126
Altered mental status (GCS ⩽ 14)	1 (4%)	4 (19%)	5 (11%)	0.102
Limb ischemia	1 (4%)	0	1 (2%)	0.354
Chest pain	0	3 (7%)	3 (7%)	0.051
Presence of distant foci of infection	3 (12%)	1 (5%)	4 (9%)^d^	0.385
Laboratory findings, mean (SD)				
Leukocytosis, cells × 10^9^/L	13.2 ± 5.4	12.1 ± 4.6	12.7 ± 5.0	0.459
Hemoglobin, g/dL	10.2 ± 2.1	10.5 ± 2.6	10.3 ± 2.0	0.699
Platelets	328 ±199	263 ± 106	299 ± 165	0.186
Acute Kidney Injury	6 (24%)	4 (19%)	10 (22%)	0.685

a Defined as the presence of suspected infection in addition to at least two SIRS criteria; Temperature > 38°C or <36°C, heart rate > 90 beats per minute, respiratory rate > 20 breath per minute, or white blood cell count of >12 × 10^9^/L or <4 × 10^9^/L.

b Presence of erythema, tenderness, edema, open wound, drainage, or sinus tract at the surgical site; in cases of combined open and endovascular repair procedures.

c Bleeding refers to hemoptysis, hematemesis, or melena.

d Discitis/osteomyelitis of the spine (*n* = 2), splenic infarction (*n* = 1), and a patient with both septic arthritis (*n* = 1) and a kidney infarction (*n* = 1).

CNS, central nervous system; EVAR, endovascular aortic repair; GCS, Glasgow coma scale; IQR, interquartile range; SD, standard deviation; SIRS, systemic inflammatory response; TEVAR, thoracic endovascular aortic repair.

Comparing overall patient characteristics between those with and without fever, no statistically significant differences were observed in underlying comorbid conditions, timing of onset of infection, WBC count at presentation, or causative organisms (Supplemental Table S1). Similarly, when comparing patients with positive versus negative blood cultures at presentation, no statistically significant differences were observed in type of graft infection, underlying immunosuppression, WBC count at presentation, or time from symptom onset to diagnosis (Supplemental Table S2).

### Imaging modalities

The most used imaging modality for diagnosing AEGI was computed tomography (CT) scan, as shown in Supplemental Table S3. All patients underwent at least one imaging modality, with several requiring multiple imaging studies to support the diagnosis. CT scan was obtained in 39 (85%) patients, a fluorodeoxyglucose (FDG) positron emission tomography-computed tomography (PET-CT) scan in 20 (43%), and an Indium-labeled tagged WBC scan in 1 (4%). Radiological findings included perigraft fluid in 15 (33%) patients, increased graft uptake in PET-CT in 13 (28%), pseudoaneurysm in 10 (22%), perigraft gas and tissue stranding in 8 (17%), perigraft abscess in 7 (15%), evidence of a fistula in 5 (11%), graft rupture or dehiscence in 3 (7%), regional lymphadenopathy in 1 (2%), and arterial graft thrombosis in 1 patient (2%); the latter being the same patient who presented with acute limb ischemia. Two patients (4%) had negative radiological findings.

### Microbiology

Blood cultures were obtained from 46 patients at the time of index admission. Positive blood cultures were identified in 25 (54%) patients; 10 (40%) patients in the EVAR group and 15 (71%) patients in the TEVAR group (*p* = 0.033). Deep tissue cultures were performed in 31 patients, of which 30 were intraoperative tissue graft cultures, and one was obtained via an interventional radiology-guided drainage of a perigraft abscess in a patient who did not undergo surgery. Among these, deep tissue cultures were positive in 20 (65%) patients; 11 (35%) in the EVAR group and 9 (29%) in the TEVAR group. The median time from symptom onset to diagnosis was 19 days (IQR 10–56) in patients with negative blood cultures compared with 9 days (IQR 4–32) in those with positive blood culture (*p* = 0.148).

*Staphylococcus aureus* was isolated in 11 (24%) patients, polymicrobial infection in 11 (24%), Enterobacterales in 6 (13%); *Escherichia coli* (*N* = 3), *Proteus* species (*N* = 2), and *Klebsiella* species (*N* = 1), Coagulase-negative staphylococci in 2 (4%), *Streptococcus* species in 3 (7%), *Pseudomonas aeruginosa* in 2 (4%), *Candida* species in 1 (2%), and other organisms listed in Table 3. In three (7%) patients, conventional microbiologic testing, including blood and tissue cultures, was negative with no causative organism identified. Plasma mcfDNA or tissue polymerase chain reaction (PCR) were not utilized in these cases.

**Table 3.** Isolated causative pathogens for AEGI.

Microbiological characteristics	EVAR, *N* = 25	TEVAR, *N* = 21	Total, *N* = 46	*p* Value
Positive blood culture	10 (40%)	15 (71%)	25 (54%)	0.033
Positive tissue culture (deep culture)^a^	11/31 (35%)	9/31 (29%)	20/31(65%)	
Pathogen causing AEGI				
*Staphylococcus aureus*	4 (16%)	7 (33%)	11 (24%)	
MRSA	4 (16%)	0	4 (9%)	
MSSA	0	7 (33%)	7 (15%)	
Polymicrobial	8 (32%)	3 (14%)	11 (24%)	
Enterobacterales	3 (12%)	3 (14%)	6 (13%)	
*E. coli*	1 (4%)	2 (10%)	3 (7%)	
*Klebsiella* species	1 (4%)	0	1 (2%)	
*Proteus* species	1 (4%)	1 (5%)	2 (4%)	
Coagulase-negative *Staphylococcus*	1 (4%)	1 (5%)	2 (4%)	
*Streptococcus species*	1 (4%)	2 (10%)	3 (7%)	
*Pseudomonas aeruginosa*	0	2 (10%)	2 (4%)	
*Candida* species	1 (4%)	0	1 (2%)	
Other organisms^b^	4 (16%)	3 (14%)	7 (15%)	
Culture negative	3 (12%)	0	3 (7%)	
Plasma mcfDNA testing utilized	3 (12%)	2(11%)	5 (11%)	
Positive result	3 (100%)	1 (50%)	4 (80%)	
Tissue universal PCR	3 (12%)	0	3 (7%)	
Positive result	2 (67%)	0	2 (67%)	

a Thirty-one refer to the total number of patients who underwent surgery or Interventional Radiology (IR)-guided drainage of a perigraft abscess.

b *Coxiella burnetii* (*n* = 1), *Haemophilus influenzae* (*n* = 1), *Lawsonella clevelandensis* (*n* = 1), *Listeria monocytogenes* (*n* = 1), *Moraxella catarrhalis* (*n* = 1), *Mycobacterium intracellulare* (*n* = 1), *Pasteurella multocida* (*n* = 1).

AEGI, aortic endograft infection; *E. coli*, *Escherichia coli*; EVAR, endovascular aortic repair; mcfDNA, microbial cell-free DNA; MRSA, methicillin-resistant *Staphylococcus aureus*; MSSA, methicillin-sensitive *Staphylococcus aureus*; PCR, polymerase chain reaction; TEVAR, thoracic endovascular aortic repair.

**Figure 2.**
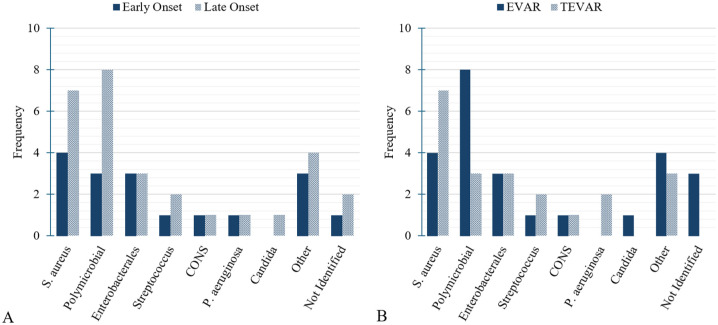
Organisms causing AEGI based on timing of infection (a), and location of the endograft (b). AEGI, aortic endograft infection.

Plasma mcfDNA test was utilized in five patients and yielded a positive result, believed to reflect the causative organism of AEGI in four patients (80%). Among these five patients, four had negative conventional blood cultures. In the remaining case, blood culture grew *P. micra* alone, while the plasma mcfDNA test identified a polymicrobial profile consisting of *P. micra*, *E. faecalis*, and *P. aeruginosa*. The pathogens causing AEGI are shown in Figure 2 and summarized in Table 3.

### Treatment and outcome

All 46 patients received antimicrobial therapy; 31 (67%) underwent surgical management. All patients with evidence of a fistula (5/5, 100%) underwent surgical graft replacement. Among those undergoing surgery, 23 (74%) had complete graft resection, 6 (19%) had partial graft resection, and 2 (7%) underwent surgical debridement without graft removal.

Of the 23 patients who underwent complete graft resection, 20 (87%) underwent in situ reconstruction, while 3 (13%) underwent extra-anatomical bypass. Among those who underwent in situ reconstruction, prosthetic grafts were used in 18 (90%) patients, of which 13/18 (72%) were rifampin-soaked Dacron grafts. Arterial allograft and xenograft were each used in 1 (5%) patient. Among those who underwent extra-anatomical bypass, prosthetic grafts were used in 2 (67%), and a venous allograft in 1 patient (33%).

Among patients undergoing surgical management, the overall median time from diagnosis to surgical intervention was 8 days (IQR 4–42).

The median duration of curative antimicrobials was 6 weeks. Curative antimicrobials used included beta-lactams in 39 (85%) patients; penicillin, cephalosporin, and carbapenem, echinocandins or azoles in 11 (24%), vancomycin in 8 (17%), rifampin in 6 (13%), daptomycin in 5 (11%), metronidazole in 4 (9%), tetracycline in 3 (7%), linezolid in 2 (4%), fluoroquinolones (FQ) in 2 (4%), and other antimicrobials in 3 (7%).

Suppressive antimicrobial therapy was used in 34 (74%) patients following completion of curative therapy; 22 (65%) of those were surgically managed, and 12 (35%) were managed medically. Suppressive therapy included tetracyclines in 15 (33%) patients, beta-lactam in 11 (24%), trimethoprim-sulfamethoxazole (TMP-SMX) in 4 (9%), azoles in 4 (9%), FQ in 3 (7%), and other antimicrobials in 2 (4%).

Treatment success was achieved in 18 (58%) patients in the surgical group compared to 9 (60%) in the medical group (*p* = 0.250). Relapsing infections occurred in 5 (16%) patients in the surgical group compared to 5 (33%) in the medical (*p* = 0.185); relapse-free survival is shown in Supplemental Figure S1. Median duration from discharge to relapse was 75 days, with most relapses occurring in less than 6 months (*n* = 9 (90%)). Only 1 (10%) patient relapsed after 6 months (Supplemental Figure S2).

The most common cause of relapse was inadequate source control, observed in 8 (80%) patients; 5 (50%) were medically managed, and 3 (30%) had partial graft resection. The remaining 2 patients (20%) had no identifiable cause for relapse, Supplemental Figure S2. Relapse cases are detailed in Supplemental Table S4. Death occurred in 8 patients (17%) during index admission with no difference among treatment groups. An additional 9 patients (20%) died during the follow-up period. Outcome of our study cohort is summarized in Table 4.

**Table 4.** Outcome of AEGI.

Treatment and outcome	Surgical and medical treatment, *N* = 31	Medical treatment alone, *N* = 15	Total, *N* = 46	*p* Value
Duration of curative antimicrobials, median	6 weeks	6 weeks	6 weeks	
Suppressive antimicrobials	22 (71%)	12 (80%)	34 (74%)	0.769
Treatment success	18 (58%)	9 (60%)	15 (33%)	0.250
Relapsed	5 (16%)	5(33%)	10 (22%)	0.185
within 3 months	4 (13%)	3 (20%)	7 (15%)	
within 3–6 months	1 (3%)	1 (7%)	2 (4%)	
After >6	0	1 (7%)	1 (2%)	
Death during index admission	5(16%)	3 (20%)	8 (17%)	0.859

## Discussion

In this study, we report interesting observations in patients presenting with AEGI. Notably, fever and bacteremia, two key clinical indicators of graft infection, were absent in approximately half of the cases. Furthermore, surgical management was associated with an overall treatment success rate comparable to that of medical therapy alone; though relapse rates were higher among patients managed non-operatively.

Over a 7-year period, 46 cases of AEGI were identified with nearly equal distribution between TEVAR and EVAR groups. Patients in the TEVAR group were younger (median age 62 years), a difference most likely explained by the underlying indication for TEVAR, such as valvular abnormalities that can affect younger patients.

Both TEVAR and EVAR groups primarily presented with late-onset AEGI (⩾3 months), as has been observed in prior studies.^15,16^ However, early-onset AEGI was more frequently observed in TEVAR compared to EVAR (Figure 1). Patients in our study cohort received TEVAR as part of ascending aortic dissection management. These patients received a complex procedure that included both an open repair with prosthetic graft placement in addition to antegrade endovascular stent deployment. Such complex procedures carry a higher risk of surgical site infection, resulting in early-onset AEGI in some of these patients. Furthermore, TEVAR patients were more likely to meet sepsis criteria, reflecting acuity of presentation.

Clinical manifestations of AEGI are heterogeneous, and diagnosing such a condition requires a high index of clinical suspicion. Fever was present in only 54% of cases, which is comparable to what has been described in the literature (59%–70%),^15,16^ indicating that absence of fever should not preclude concern for underlying AEGI diagnosis. Other presenting symptoms were non-specific, including fatigue, nausea, vomiting, or generally not feeling well. Focal symptoms such as abdominal pain or back pain might help localize the infection of the abdominal graft, in contrast to chest pain, which might help localize the infection to the thoracic graft. Gastrointestinal bleeding or hemoptysis, particularly in late-onset AEGI, should raise the concern of aorto-enteric and aorto-bronchial fistula.

Given the non-specific symptoms, imaging is crucial for diagnosis. Among available modalities, WBC scintigraphy with SPECT/CT is the best modality for diagnosis, with a sensitivity of 99% and 82% specificity, followed by FDG-PET/CT and then CT angiography (CTA) scan.^17^ Most of the patients in our study cohort underwent CTA scan as an initial imaging modality because of its widespread availability, rapid acquisition time, and minimal preparation requirements compared to FDG-PET/CT and WBC Scintigraphy. FDG-PET/CT was typically performed as a secondary modality if initial imaging was inconclusive. In one case, an Indium-labeled leukocyte scan was obtained due to diagnostic uncertainty, as it remains the most specific modality. This approach aligns with the recommendation of the European Society for Vascular Surgery (ESVS), European Association for Cardiothoracic Surgery (EACTS), the Society of Thoracic Surgeons (STS), and the American Heart Association (AHA).^8,9^ In some settings, where FDG-PET/CT is readily available, it may be considered a preferred initial imaging modality given its superior sensitivity compared to CTA scans. In our cohort, the most common imaging findings were the presence of perigraft fluid and increased graft uptake.

Blood cultures were positive in only half of the total cohort. However, subgroup analysis revealed a higher rate of positivity in the TEVAR group compared to EVAR. This may be related to the baseline characteristics of the TEVAR group and the underlying indications for TEVAR placement, as previously discussed. Intraoperative cultures were positive in 65% of those who underwent surgery. Both blood and intraoperative culture positivity rates were lower than previously reported by Smeds et al.,^15^ who found 63% and 72% positivity rates, respectively.

This overall low rate of blood culture positivity may be attributed to a low burden of organisms and widespread use of antimicrobials for various indications in this patient population. It is plausible that some patients had received antimicrobials in months prior to diagnosis, as their initial presentation was often non-specific and may have been attributed to other infections. While this cannot be confirmed in our study, it is a reasonable explanation for the reduced sensitivity of blood cultures. In addition, median time to surgery in our cohort was 8 days, indicating that most patients received antimicrobials for at least a week before intraoperative cultures were obtained. This likely contributed to the lower intraoperative culture positivity rate.

Considering that blood cultures remain negative in almost half of the patients with AEGI, next-generation plasma mcfDNA sequencing may be a useful adjunctive tool to detect microbiologic etiology in these cases with negative blood cultures.^18^ In our cohort, plasma mcfDNA (Karius^®^ test) was used in five patients, four with negative blood cultures and one with incomplete polymicrobial identification. The test was positive in four patients (80%), which helped guide antimicrobial therapy. Despite its current cost, the use of this test may be justified in the context of high morbidity and mortality associated with AEGI and the need for identification of causative pathogens to guide therapy.

All patients received prolonged targeted antimicrobial therapy, with the majority continuing suppressive therapy afterward. The use of CAS therapy, when prescribed, was not interpreted as evidence of persistent infection, but instead reflected variability in clinical practice and the absence of standardized guidance for long-term management.

The majority of patients in our cohort underwent complete surgical graft explantation with in situ reconstruction using a prosthetic graft, of which 72% were rifampin-soaked Dacron grafts. The use of prosthetic grafts was driven by practical and patient-specific considerations, particularly the frequent size and length mismatch between available autologous conduits and the aortic segment requiring reconstruction. Although cryopreserved allografts have been associated with lower reinfection risk in prior studies, comparative data demonstrate similar 5-year freedom from reinfection between rifampin-soaked prosthetic grafts and allografts (87.2% vs 94.9%), with a tendency toward higher reoperation rates in the allograft group.^19^ Rifampin-soaked Dacron is a reasonable option alongside alternatives such as autologous femoral vein and cryopreserved allografts; however, conduit selection ultimately requires balancing infection control, durability, and operative risk.

On reviewing potential factors that influenced the decision between medical and surgical management of graft infections, the only consistent pattern was that all patients with evidence of a fistula underwent surgical graft replacement. This suggests that the presence of a fistula may be a decisive factor, prompting surgical intervention. Relapses were numerically lower in the surgical group compared to those who were only treated medically, although the difference was not statistically significant. Treatment success rates were similar between groups, with an overall mortality of 17%. These findings differ from those reported in prior literature, which reported a mortality of 80% in those who were managed non-surgically in comparison to 51% in those treated surgically.^15^ However, their medically managed group was only nine patients, who may have been sicker at baseline and therefore deemed not candidates for open surgery. This may have contributed to the higher observed mortality in those managed non-surgically. Despite the inherent morbidity and mortality associated with open surgical repair for endograft infections, particularly if delayed,^20^ we believe surgical management offers better long-term outcomes. The lack of statistically significant differences in our cohort may reflect limitations in sample size.

We found no association between causative organisms and either mortality or relapse rate. In addition, there was no association between the class of antimicrobials used, whether for curative or suppressive treatment, and clinical outcome. This is contrary to what was described in a prior study where polymicrobial and gram-negative infections had decreased survival.^15^ Furthermore, we were unable to evaluate differences in outcome and relapse based on the type of graft used for aortic reconstruction, as most patients underwent prosthetic reconstruction with Dacron grafts, with very limited use of cryopreserved or venous allografts, and no use of neo-aortoiliac system (NAIS) reconstruction in our cohort, precluding meaningful comparison.

The current study has several limitations, primarily due to its retrospective design and BSLMC being a tertiary referral center. The absence of a specific ICD-10 code for AEGI may have limited our case identification during the screening process. Furthermore, as a referral center for the surrounding cities and states, we could not ascertain the total number of endograft procedures performed, precluding incidence calculation. In addition, data regarding prehospital antimicrobials use were often unavailable. Finally, given the rarity of AEGI, small sample size, and event rates limited our ability to conduct statistical analyses other than a descriptive evaluation.

## Conclusion

Most patients in our study cohort presented with late-onset AEGI. Fever was absent in nearly 40% of cases, and blood cultures were negative in almost half, highlighting the need for a high clinical suspicion. While surgical and medical management demonstrated similar treatment success and mortality rates, relapses were more frequent in the medically treated group. These findings should be interpreted with caution as limited sample size, potential selection bias, and lack of long-term follow-up may obscure true differences between management strategies. Further studies with larger cohorts and longer follow-up are warranted to elucidate optimal management strategies.

## Supplemental Material

sj-docx-1-tai-10.1177_20499361261465761 – Supplemental material for Clinical presentation, management, and outcomes of aortic endograft infections: a retrospective descriptive study Supplemental material, sj-docx-1-tai-10.1177_20499361261465761 for Clinical presentation, management, and outcomes of aortic endograft infections: a retrospective descriptive study by Haitham Alaithan, Sarwat Khalil, Gustavo Oderich, Larry M. Baddour and Muhammad Rizwan Sohail in Therapeutic Advances in Infectious Disease

sj-pdf-1-tai-10.1177_20499361261465761 – Supplemental material for Clinical presentation, management, and outcomes of aortic endograft infections: a retrospective descriptive study Supplemental material, sj-pdf-1-tai-10.1177_20499361261465761 for Clinical presentation, management, and outcomes of aortic endograft infections: a retrospective descriptive study by Haitham Alaithan, Sarwat Khalil, Gustavo Oderich, Larry M. Baddour and Muhammad Rizwan Sohail in Therapeutic Advances in Infectious Disease

## Declarations

**Ethics approval and consent to participate:** This study was approved by the Baylor College of Medicine Institutional Review Board (IRB Protocol H-56084), which granted a waiver of informed consent due to the retrospective nature of the research and minimal risk to participants.

**Consent for publication:** Not applicable.

**Availability of data and materials:** The data supporting the findings of this study are provided in the Supplemental Files accompanying this manuscript.
