# Supplementary material for: Clinical presentation, management, and outcomes of aortic endograft infections: a retrospective descriptive study
Source: Ther Adv Infect Dis. 2026 Jul 16;13:20499361261465761. doi: 10.1177/20499361261465761 (PMC13376498; doi:10.1177/20499361261465761)
Supplement: sj-docx-1-tai-10.1177_20499361261465761 – Supplemental material for Clinical presentation, management, and outcomes of aortic endograft infections: a retrospective descriptive study [file sj-docx-1-tai-10.1177_20499361261465761.docx]

**Supplementary Table S1. Comparison of Study Cohort Characteristics Based on Presence or Absence of Fever**

| Patient Characteristics | No Fever **N=21** | Fever  **N=25** | P-value |
| --- | --- | --- | --- |
| Age, y, median (IQR) | 65 (61-74) | 64 (61-72) | 0.856 |
| Charlson comorbidity index score, SD | 4 ±2 | 3 ± 2 | 0.326 |
| Immunocompromised (presence of any immunocompromised condition) | 2 (10%) | 2 (8%) | 1.000 |
| Type of Graft |  |  | 0.387 |
| EVAR | 13 (52%) | 12 (48%) |  |
| TEVAR | 8 (38%) | 13 (62%) |  |
| Onset of Infection |  |  | 0.260 |
| Early | 7 (41%) | 10 (59%) |  |
| Late | 14 (48%) | 15 (52%) |  |
| Labs |  |  |  |
| Leukocyte, cells x 10^9^ | 12 ± 5 | 13 ± 4 | 0.573 |
| Organism |  |  |  |
| Gram positive | 7(39%) | 11(61%) | 0.752^a^ |
| Gram negative | 6 (50%) | 6(50%) | 0.734^a^ |
| Polymicrobial | 5(45%) | 6(54%) | 1.000^a^ |
| Positive Blood culture | 10 (40%) | 15 (60%) | 0.401 |
| Abbreviations: IQR, interquartile range. SD, standard deviation  ^a^ P values represent Fisher’s exact test comparing each organism group (Gram positive, Gram negative, or Polymicrobial) with all other organisms for the presence of fever. | | | |

**Supplementary Table S2. Comparison of Study Cohort Characteristics Based on Blood Culture Results**

| Patient Characteristics | Negative Blood culture  **N=21** | Positive Blood Culture  **N=25** | P-value |
| --- | --- | --- | --- |
| Age, y, median (IQR) | 65 (61-74) | 64 (60-72) | 0.748 |
| Type of Graft |  |  | **0.033** |
| EVAR | 15 (60%) | 10 (40%) |  |
| TEVAR | 6 (29%) | 15 (71%) |  |
| Immunocompromised^a^ | 2 (50%) | 2 (50%) | 1.000 |
| Labs |  |  |  |
| Leukocyte, cells x 10^9^ | 12.6 ± 5 | 13 ± 5 | 0.714 |
| Time from symptoms to diagnosis, days, median (IQR) | 19 (IQR 10-56) | 9 (IQR 4-32) | 0.148 |
| Abbreviations: IQR, interquartile range  ^a^ Immunocompromised refers to patients with immunocompromised conditions including receiving chemotherapy, immunotherapy, cirrhosis, uncontrolled human immunodeficiency virus (HIV), high dose steroids. | | | |

**Supplementary Table S3. Comparison of Imaging Modalities and Radiological Findings in EVAR vs. TEVAR in Our Study Cohort**^a^

|  | EVAR  N=25 | TEVAR  N=21 | Total  N=46 | P value |
| --- | --- | --- | --- | --- |
| Modalities used for diagnosis |  |  |  |  |
| CT scan | 20 (80%) | 19 (90%) | 39 (85%) | 0.324 |
| PET Scan | 12 (48%) | 8 (38%) | 20 (43%) | 0.500 |
| WBC Tagged scan | 1 (4%) | 0 | 1 (2%) | 0.354 |
| Radiological findings, at graft site |  |  |  |  |
| Perigraft fluid | 8 (32%) | 7 (33%) | 15 (33%) | 0.923 |
| Increased uptake in PET scan | 8 (32%) | 5 (24%) | 13 (28%) | 0.539 |
| Pseudoaneurysm | 7 (28%) | 3 (14%) | 10 (22%) | 0.306 |
| Tissue stranding | 6 (24%) | 2 (10%) | 8 (17%) | 0.197 |
| Peri-graft gas | 3 (12%) | 5 (24%) | 8 (17%) | 0.439 |
| Abscess | 5 (20%) | 2 (10%) | 7 (15%) | 0.428 |
| Sinus tract or fistula | 3 (12%) | 2 (10%) | 5 (11%) | 1.000 |
| Bleeding (graft rupture or dehiscence) | 2 (8%) | 1 (5%) | 3 (7%) | 1.000 |
| Regional lymphadenopathy | 1 (4%) | 0 | 1 (2%) | 1.000 |
| Arterial graft thrombosis | 1 (4%) | 0 | 1 (2%) | 1.000 |
| Evidence of distant foci of infection | 3 (12%) | 1 (5%) | 4 (9%)^b^ | 0.614 |
| Abbreviations: Computed tomography (CT), magnetic resonance imaging (MRI), positron emission tomography (PET), white blood cell (WBC)  ^a^ Percentages do not total 100% as some patients underwent multiple imaging modalities for the diagnosis of AEGI  ^b^ Discitis/osteomyelitis of the spine (n=2), splenic infarction (n=1), and a patient with both septic arthritis (n=1), and a kidney infarction (n=1) | | | | |

**Supplementary Table S4. Clinical Characteristics of Patients with Relapsed Infection in Our Study Cohort (n=10)**

| **Relapse** | **Endograft location** | **Onset of infection^a^** | **Organism** | **Distant Foci** | **Prosthetic material** | **Surgical graft resection** | **Duration of therapeutic antimicrobials** | **Suppressive antimicrobials** | **Timing of relapse** | **Reason for relapse** | **Outcome** |
| --- | --- | --- | --- | --- | --- | --- | --- | --- | --- | --- | --- |
| **1** | TEVAR | Late | Polymicrobial | - | - | Partial | 6 weeks IV | - | ≤3 months | Incomplete graft resection | Death |
| **2** | TEVAR | Early | H. Influenza | - | - | Complete | 3.5 months IV | - | ≤3 months | Unclear | Death |
| **3** | TEVAR | Late | S. aureus | - | Prosthetic heart valve | Partial | 6 weeks IV | Tetracyclines | 3-6 months | Incomplete graft resection | Death, unclear if related to AEGI |
| **4** | TEVAR | Late | S. aureus | - | Prosthetic Joint | Partial | 6 weeks IV | Tetracyclines | ≤3 months | Incomplete graft resection | Readmission: I&D of mediastinal/perigraft collection, lifelong suppression |
| **5** | TEVAR | Late | P. aeruginosa | - | - | No surgery | 6 weeks IV | FQ | >6 months | Inadequate source control | Readmission: declined surgery, cefepime changed to meropenem |
| **6** | EVAR | Early | S. aureus | Spine, OM | - | No surgery | 6 weeks IV | Tetracyclines | ≤3 months | Inadequate source control | Readmission: not surgical candidate, hospice care |
| **7** | EVAR | Late | C. burnetii | Spine, OM | Prosthetic Joint | Complete | 2 years oral | Tetracyclines, hydroxychloroquine | ≤3 months | Unclear, possible nature of disease | Death |
| **8** | EVAR | Early | E. coli | - | - | No surgery | 6 weeks IV | Tetracyclines | ≤3 months | Inadequate source control | Readmission: aneurysm rupture, complete graft explant with in-situ reconstruction, ongoing antimicrobial therapy |
| **9** | EVAR | Late | Proteus species | - | Prosthetic vascular graft | No surgery | 6 weeks IV | Cefdinir | ≤3 months | Inadequate source control | Readmission: partial graft explant, ongoing antimicrobial therapy, followed by suppression |
| **10** | EVAR | Early | Lawsonella clevelandensis | - | - | No surgery | 6 Weeks IV | Tetracyclines | 3-6 months | Inadequate source control | Readmission: complete graft explant with in-situ reconstruction, LTACH |
| Abbreviations: Incision and Drainage (I&D), Long term acute care hospital (LTACH)  ^a^ Early onset infection (<3 months after endograft placement), Late onset infection (≥ 3 months) | | | | | | | | | | | |

**Supplementary Figure S1.** Probability of Relapse-Free-Survival (number of events = 10)


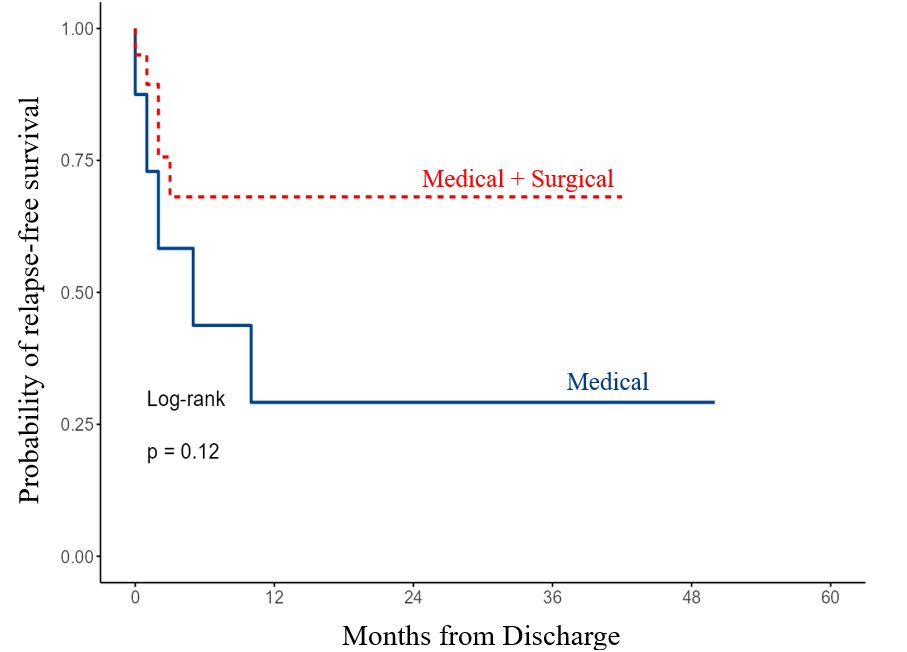


**Supplementary Figure S2.**  Timing of relapse after index admission (A), and reasons for relapse (B)
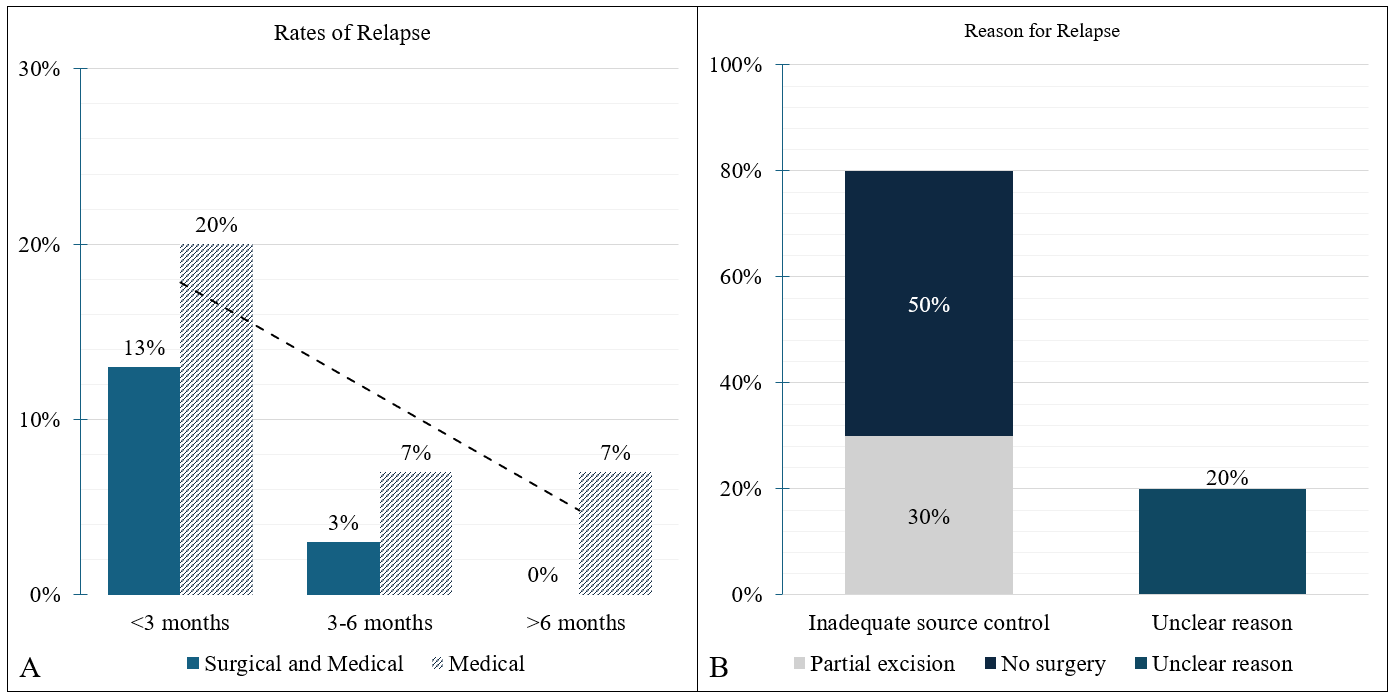


**Alt text.** Two bar charts. The chart on the left, labeled A, shows the rate of relapses based on the timing of relapses, in months. The chart on the right, labeled B, shows the reasons for relapses
